# Black male coaches: an intersectional analysis of their contributions to positive youth development

**DOI:** 10.3389/fpsyg.2026.1815811

**Published:** 2026-06-22

**Authors:** Mitchell Allen, Isaac Woods, Jarrett Murphy

**Affiliations:** 1 Department of Education, School, & Counseling Psychology, University of Kentucky, Lexington, KY, United States; 2 Department of Psychology, North Carolina State University, Raleigh, NC, United States; 3 Department of Educational Studies, The Ohio State University, Columbus, OH, United States

**Keywords:** Black boys, Black men, Black psychology, mental health, positive youth development, prevention, sports

## Abstract

**Introduction:**

Black male educators are underrepresented in U.S. schools, comprising less than 2% of the teaching workforce. That leaves Black boys critically underserved despite their growing population and documented need for Black male role models and mentors. Black boys face disproportionate risks for both homicide and suicide, underscoring the urgency of identifying community-based supports that promote their wellbeing.

**Methods:**

This qualitative study employed semi-structured interviews with five Black male sports coaches serving youth across various regions of the United States. Intersectionality theory and the Phenomenological Variant of Ecological Systems Theory (PVEST) guided the study’s design and analytical approach. Data were analyzed using thematic analysis.

**Results:**

Five major themes emerged from the data: Community Pillars, No Limits to Coaching, Relationship Fundamentals, Societal Privilege and Power Dynamics, and Struggles. Findings demonstrate that Black male coaches leverage their intersectional social position and relationships with Black boys to promote positive youth development, identify mental health concerns, and provide emotional support. Coaches functioned as vital social capital within Black communities, serving in roles as social fathers, advocates, and informal mental health supports.

**Discussion:**

This research advances understanding of how Black male coaches contribute to positive youth development and suicide prevention efforts among Black youth. Implications are discussed for Black psychology, educational policy, and the design of community-based interventions.

## Introduction

NFL player Travis Hunter once expressed that, “it’s a lot that [Coach Prime] has done for me outside of football. He’s like a father figure to me. I can go to him for anything” (Marca, 2025). These words illuminate a reality often overlooked in sports, which is that Black male coaches often possess a unique social capital, and function as far more than just athletic trainers. The importance of Black male representation in child-care service professions, as well as in the role of fathers, cannot be overstated. In a society where stereotypes and biases often marginalize Black men, their presence in caregiving roles serves as a powerful counter-narrative that challenges harmful stereotypes and promotes positive role modeling (Brown and Thomas, 2020; Curry, 2018). Black fathers play crucial roles in shaping the emotional and social development of their children, including aiding in the development of adaptive coping skills for discriminatory encounters and racialized contexts (Cooper J. N. et al., 2020; Cooper S. M. et al., 2020). Mainstream institutions offer few culturally responsive mentoring structures or community-based support systems specifically designed to equip Black boys with strategies for negotiating racism, violence, and structural barriers (Sánchez et al., 2017). By actively engaging in caregiving activities and nurturing relationships with their children, Black fathers defy societal norms and demonstrate the importance of hands-on involvement in parenting.

Building on this foundation, Black male representation in child-care professions such as teaching, counseling, social work, and sports coaching not only provides essential support and guidance to children in need but also offers young Black boys valuable role models to look up to (Brown and Thomas, 2020; Bristol and Goings, 2019). Research demonstrates that Black students who have at least one Black teacher in elementary school are more likely to enroll in college, with this effect increasing after having two Black teachers (Gershenson et al., 2022). The presence of Black male educators has been shown to reduce dropout rates, decrease disciplinary actions, and increase college aspirations among Black male students specifically (Lindsay and Hart, 2017; Mee et al., 2024). Ultimately, the presence of Black men in caregiving professions and as fathers catalyzes positive social change, fostering stronger families, healthier communities, and a more inclusive society.

Black male coaches often serve as more than just athletic trainers as they function as social fathers within their communities (Richardson, 2012). In many cases, these coaches provide guidance, support, and mentorship to young athletes beyond the playing field (Gould et al., 2007). These coaches draw from their own experiences navigating systemic barriers and racial inequalities, offering invaluable insights and encouragement to their players while serving as positive role models and sources of inspiration (Richardson, 2012; Thomas, 2023). Through their leadership, Black male coaches instill important values such as resilience, discipline, and perseverance, helping to shape not only the athletic abilities of their players but also their character and sense of identity while providing safe and supportive spaces for personal growth and development (Gould et al., 2007; Richardson, 2012).

The focus on Black males in the present study reflects the distinct and compounding set of risks and resource deficits that characterize the developmental context of Black boys in the United States. Black male students are disproportionately subjected to punitive school discipline, including suspension and expulsion at rates approximately three times higher than their White peers, a pattern that systematically funnels Black boys toward the school-to-prison pipeline rather than toward supportive educational experiences (Jeter and Melendez, 2022; Marraccini et al., 2023). This intersection of heightened systemic exposure to marginalization and the systematic absence of the very supports most likely to buffer its effects positions Black boys as a uniquely underserved population, and renders focused scholarly attention to the adults who organically fill these relational gaps. The current literature remains limited in its focus on Black male coaches and their contributions to the communities they serve, as well as to understanding those who serve as Black male coaches and educators in school systems (Thomas, 2022). The development of teacher-coach literature has predominantly been conducted within a White racial framework, underscoring the importance of investigating the experiences and salient impact of Black male coaches (Thomas, 2023). Thomas (2023) documented how the Black history of teacher-coaches is historically separate and distinct from the American history of teacher-coaches, rooted in traditions of resistance to anti-Blackness through intellectual thought and athletics while creating spaces of civic engagement. This finding becomes increasingly alarming considering that less than 2% of the United States teaching population is comprised of Black men (Jeter and Melendez, 2022). With low numbers of Black men occupying roles such as educators, psychologists, psychiatrists, and pediatricians, Black youth are unlikely to be served by Black men in these professional roles.

In fact, it is more probable that Black youth will have a Black male coach than a Black male educator, psychologist, psychiatrist, or pediatrician. Black male coaches often assume various supportive roles outside the typical confines of leading their team on the field. Richardson (2012) illuminates how coaches serve as valuable social capital for Black youth and the Black community. The presence and active engagement of Black male coaches can serve as a protective factor and informal social support within Black communities, contributing to safety for Black youth, particularly in areas where traditional adult Black male figures are lacking due to factors like unemployment, violence, systemic barriers, and high rates of incarceration among Black men (Congressional Black Caucus Emergency Task Force on Black Youth Suicide and Mental Health, 2019; Richardson, 2012). Woods et al. (2023) further documented how Black male coaches can be leveraged to promote community wellness, health, and academic success for Black youth, noting that previous research has demonstrated several positive outcomes including academics, social skills, and reducing misbehaviors when the racial identity of Black students is matched with a Black coach. This study explores how Black male sports coaches leverage their unique intersectional position to promote positive youth development, identify mental health concerns, and serve as community advocates for the Black youth they serve.

In the current study, we review relevant literature on positive youth development through sport, Black male educators and mentorships, the Black youth mental health crisis, and coach-athlete relationships to establish the theoretical and empirical foundation for our study. Second, present our conceptual framework integrating intersectionality theory and PVEST, explaining how these lenses illuminate Black Black male coaches’ unique positioning. Third, describe our qualitative methodology, including our semi-structured interview approach, participant recruitment and thematic analysis procedures. Fourth, we present our five major themes with rich illustrative quotes from our participant coaches. Finally, we discuss theoretical and practical implications, situating our findings within scholarship while advancing new conceptualizations of Black male coaches as intersectional cultural brokers whose work extends far beyond athletic instruction to encompass mental health support, community advocacy, and youth empowerment.

## Literature review

### Positive youth development through sport

Positive Youth Development (PYD) represents an asset-building approach emphasizing enhancing strengths and developing potential in all youth (Holt and McDonough, 2024). Sport has been identified as a particularly promising context for positive youth development due to its widespread appeal, opportunity for exercise, structured nature, and opportunities for meaningful adult-youth relationships (Bruner et al., 2023; Vella et al., 2011). A comprehensive qualitative meta-study by Holt et al. (2017) analyzed 66 studies to develop a grounded theory of positive youth development through sport, identifying three core themes consisting of positive youth development climate involving supportive social contexts, life skills program focus involving intentional teaching strategies, and positive youth development outcomes involving competencies developed. Critically, their findings revealed that transfer of skills from sport to other life domains requires explicit pedagogical strategies from coaches rather than occurring automatically through sport participation alone.

Recent meta-analytic evidence suggests sport-based interventions show promise for promoting select developmental outcomes, though methodological limitations in the existing literature warrant caution in interpretation (Bruner et al., 2023). However, researchers emphasize that outcomes depend on what works, for whom, and in what circumstances. Bruner et al.’s (2023) meta-analysis examining sport-based positive youth development interventions found meaningful positive effects on young people’s sense of competence, confidence, and life skills development. Programs such as Girls on the Run, the First Tee Program, and the LiFE Sports Camp were consistently associated with favorable developmental outcomes across multiple studies. However, the authors noted that effects on character development, social connection, health outcomes, and overall PYD climate were less consistent across the literature.

Li et al. (2025) conducted a systematic review and meta-analysis on the impact of coach education on coaching effectiveness in youth sport, finding that coach training participation significantly predicts greater perceived impact on athletes’ life skills development. Coach training in life skills development predicts greater perceived impact on athletes, with prior training in mental health also associated with coach satisfaction and success (Anderson-Butcher et al., 2025). Studies examining award-winning high school coaches found that effective coaches have clear philosophies prioritizing personal development and use strategies such as sharing personal reflections, establishing behavioral contracts, organizing team-building activities, and explicitly teaching life skills (Gould et al., 2007).

Despite this body of research, several critical gaps remain. Most positive youth development through sport research has been conducted with White coaches and athletes, limiting understanding of how race, culture, and intersectional identities shape coaching practices and outcomes. While coach-athlete relationships are recognized as foundational to positive youth development (Jowett, 2017), few studies examine how coaches from marginalized communities leverage their lived experiences and cultural knowledge to promote development (Whitley et al., 2019). Although mental health has emerged as a critical concern in youth sport, research on coaches’ roles in identifying and supporting youth mental health needs remains limited.

### Black male educators and mentorship

A substantial body of research demonstrates that Black male educators significantly impact Black students’ academic achievement, school engagement, and long-term outcomes. Gershenson et al. (2022) found that having just one Black teacher in elementary school increased high school graduation rates for Black students by 13% and college enrollment by 19% compared to Black students who did not have a Black teacher. For Black male students from low-income households, effects were even more pronounced. Lindsay and Hart (2017) documented that exposure to Black teachers reduced disciplinary actions for Black students, while Hart (2020) showed increased enrollment in honors courses. These effects persist across multiple studies using different methodologies and datasets (Egalite et al., 2015; Mee et al., 2024).

Research suggests several mechanisms underlying these positive effects. Black teachers often hold higher expectations for Black students, employ culturally responsive pedagogies, and serve as relatable role models (Blazar, 2024; Bristol, 2020). They are better positioned to recognize and validate students’ experiences with microaggressions and systemic racism while teaching adaptive coping strategies (Thomas, 2022). Additionally, Black male educators often view their work through a social justice lens, seeing teaching as a means of community uplift and racial equity advancement (Brown and Thomas, 2020). Thomas (2022) found that Black male teacher-coaches often engage in unique, civic-oriented practices in their teaching and coaching that counter historical anti-Black narratives in education and society, demonstrating the range of intellectual, pedagogical, and ideological discourses amongst Black men that extends far beyond the problem narrative that stereotypically oversimplifies Black male teachers.

Despite documented benefits, Black male educators remain severely underrepresented, comprising less than 2% of the public school teaching force (Jeter and Melendez, 2022). Multiple barriers contribute to this shortage, including restrictive certification requirements, lack of recruitment pathways, unwelcoming school climates, and limited support structures (Bristol and Goings, 2019). Consequently, most Black boys complete their entire K-12 education without ever having a Black male teacher. Formal mentoring programs targeting Black male youth have shown promise in addressing this gap. Programs explicitly designed for boys and young men of color, incorporating culturally responsive practices and addressing systemic barriers, demonstrate particularly strong effects (MENTOR and My Brother's Keeper Alliance, 2022).

### Black youth mental health crisis

Black youth face a mental health crisis that demands urgent attention. From 2001 to 2015, suicide rates for Black children aged 5–11 were approximately twice those of White children of the same age (Bridge et al., 2018). Among Black youth aged 10–14, death by suicide increased by 131.5% over two decades (Meza et al., 2022). From 2003 to 2017, Black youth experienced significant upward trends in suicide with the largest annual percentage changes among 15-17-year-olds including girls (Sheftall et al., 2022). The Centers for Disease Control and Prevention reported a concerning significant increase in suicide rates among Black youth aged 10 to 24 from 2018 to 2021 (Stone et al., 2023).

These alarming trends occur within a context of significant mental health care disparities. Black adolescents are significantly less likely than peers in other demographic groups to receive mental health care due to systemic inequities including racism and poverty, deeply rooted stigma around mental health, and well-founded cultural mistrust of the health care system (Lindsey et al., 2013). Research by Ballesteros and Tran (2020) found that 78% of racial and ethnic minority student athletes reported some form of mental health need, yet less than 11% of those athletes reported using mental health services in the past year, highlighting the significant gap between need and service utilization. When Black youth do access services, they face provider bias, underrepresentation of multicultural perspectives, and misdiagnosis (Burkett, 2017).

Risk factors for Black youth suicide include experiences with racism and discrimination, racial disparities in mental health care utilization, poverty, trauma, violence, and exposure to death among Black individuals in their neighborhoods (Sheftall et al., 2022). The Congressional Black Caucus Emergency Task Force on Black Youth Suicide and Mental Health (2019) identified structural inequities as a fundamental cause of mental health disparities, calling for comprehensive prevention efforts that address upstream social determinants of health. Protective factors include strong cultural identity, connectedness to community, and access to culturally responsive support systems (Meza et al., 2022).

Schools have been identified as promising settings for suicide prevention; however, Black youth may feel uncomfortable disclosing suicidal ideation in these settings due to fear of disciplinary action given longstanding racial disparities in school discipline (Sheftall et al., 2022). Community-based settings such as community centers, Boys and Girls Clubs, after-school programs, and faith-based organizations may provide more culturally safe environments for suicide prevention efforts (Congressional Black Caucus Emergency Task Force on Black Youth Suicide and Mental Health, 2019). This suggests that Black male coaches, who often operate in these community spaces and have established trusting relationships with youth, may be uniquely positioned to support mental health and suicide prevention efforts.

Williams (2023) documented the mental health struggles tied to Black masculinity among Black athletes, highlighting the complicated nature of mental health struggles and how suicide is handled in the sports world with juxtaposed racial frameworks. Williams revealed how Black male athletes often internalize hypermasculine expectations that discourage help-seeking, with one athlete disclosing that as Black men, nobody teaches them how to navigate dark thoughts. The athlete expressed that hearing others openly discuss mental health struggles at an academic conference allowed him to finally voice experiences he had been suppressing, and he later thanked Williams for showing him he was not crazy for having such thoughts. Williams argued that coaches, mentors, and administrators must intentionally develop environments where Black athletes can voice their struggles without fear of being perceived as weak, lazy, or difficult.

The mental health of Black student athletes represents a particularly understudied area. Wilkerson et al. (2020) examined Black football student-athletes’ barriers to seeking mental health services, finding that pursuing mental health services or even discussing mental health was viewed as a weakness, symbolizing the instability and stigmas often associated with mental health. Participants revealed that the silence, or the absence of conversations about mental health, reflected a lack of awareness regarding mental health services. Athletic culture instructs men to be strong and healthy and to walk it off and fight through it, and this mentality continues off the field and can impede efforts to receive mental health services.

### Coach-athlete relationships

The coach-athlete relationship has been recognized as foundational to both athletic performance and athlete wellbeing (Jowett, 2017). Jowett’s 3 + 1Cs model conceptualizes quality relationships as characterized by Closeness involving mutual feelings of trust and respect, Commitment involving long-term orientation to relationship, Complementarity involving cooperative interactions, and Co-orientation involving shared understanding of relationships. Research demonstrates that high-quality coach-athlete relationships predict numerous positive outcomes including athlete satisfaction, self-confidence, psychological wellbeing, performance, and reduced burnout (Davis and Jowett, 2014).

From a social capital perspective, coaches serve as bridges connecting youth to resources, opportunities, and supportive networks (Welty Peachey et al., 2018). Richardson’s (2012) seminal work demonstrated that Black male coaches function as valuable social capital for inner-city African American males, providing guidance that extends far beyond athletic instruction. However, limited research has examined how Black male coaches specifically navigate their intersectional identities to build relationships and promote development, particularly regarding mental health support. Cooper J. N. et al. (2020) and Cooper S. M. et al. (2020) called for culturally responsive transformational leadership in college sports, arguing that leaders who adopt culturally responsive and multicultural orientations can better address systemic inequities. They noted that Black coaches are less likely to be rehired once fired even when they have better or comparable win-loss records compared to their White peers, reflecting differential coaching expectations based on race.

### Current research gaps and study significance

Despite substantial research on positive youth development through sports, Black male educators, youth mental health, and coach-athlete relationships, critical gaps remain at the intersection of these domains. There is limited research focused on Black male coaches specifically. While research documents the benefits of Black male educators broadly, few studies focus specifically on Black male coaches. Furthermore, there is a dearth of examination of mental health support roles. Although coaches are increasingly recognized as important adult support for youth mental health, limited research examines how coaches identify and respond to mental health concerns. Additionally, there has been limited application of culturally relevant theoretical frameworks. Most coaching research employs theoretical frameworks developed without attention to race, culture, or intersectionality.

Moreover, the gap remains due to the lack of attention to community-level contributions. Richardson (2012) identified this gap over a decade ago, noting that despite considerable attention to African-American boys’ challenges in using sports to escape poverty, little attention has been devoted to understanding the unique relationship between players and coaches, and that very little documented evidence in the sociological literature explores the relationship of player and coach both on and off the field. This study addresses identified gaps by exploring how Black male coaches promote positive youth development among the Black youth they serve and what their experiences are in identifying and supporting the social, emotional, and behavioral needs of Black youth.

## Conceptual framework

### Intersectionality theory

Crenshaw (1998) coined the term intersectionality to acknowledge how various aspects of an individual’s identity, such as race and ethnicity, gender, class, sexuality, ability status, and other social categories, intersect and interact with each other to shape experiences of power and oppression. These interactions occur within the context of socio-political systems and structures of power, which often produce unique experiences of discrimination, privilege, and marginalization (Hankivsky, 2014). Intersectionality recognizes that these different dimensions of identity do not exist solely as mono-categories but instead intersect to shape individuals’ lived experiences and social outcomes.

Through an intersectional lens, Black male coaches embody a convergence of identities, experiences, and societal structures that influence their roles within the athletic coaching realm. For Black male coaches, intersectionality means navigating the complex interplay between race and gender while operating within sports programs whose resources are governed by socio-economic systems and the predominantly White, male-dominated realm of coaching (Norman, 2021; Thomas, 2023). Black male coaches simultaneously experience marginalization due to their racial identity while potentially accessing privileges associated with masculinity and athletic achievement.

Empirical applications of intersectionality in sport contexts demonstrate how coaches with marginalized identities navigate oppressive systems while leveraging their positioning to support athletes (Norman, 2021). Black male coaches are positioned at the intersection of being educators, mentors, and athletic coaches (Thomas, 2022). They use sports as a conduit to address school-related issues, community and civic duties, psychological wellbeing, issues surrounding race, interpersonal relationships, and provide many other pedagogical benefits (Thomas, 2022, 2025). Their lived experiences with racism, discrimination, and systemic barriers equip them with cultural knowledge and adaptive coping strategies they can transmit to youth facing similar challenges (Brown and Thomas, 2020). Thomas (2025) further documented how Black male teacher-coaches leverage Black counterpublics in their work with youth, drawing on the Black family as a source of strength, embracing liberatory visions, and resisting narrow constructions of Blackness.

### Phenomenological variant of ecological systems theory

To conceptualize the multifaceted roles Black male coaches occupy, our study grounds itself in the Phenomenological Variant of Ecological Systems Theory or PVEST (Spencer et al., 1997). The PVEST model was designed to examine person-processes-context relationships, specifically how Black adolescents’ perspectives on the world interact with socio-cultural and historical forces, which in turn shape individual development. Unlike traditional ecological models that may inadequately account for experiences of marginalized groups, PVEST explicitly centers the phenomenological experiences of individuals with marginalized identities navigating oppressive systems.

PVEST consists of five interconnected components. The first component involves risk contributors, which are challenges stemming from societal position, including responses to racism, discrimination, and biases. The second component involves stress engagement, which encompasses actual experiences of stress resulting from risk contributors. The third component involves reactive coping methods, which are immediate responses to stress that may be effective or ineffective. The fourth component involves stable coping responses or emergent identities, which emphasizes the importance of ethnic and racial identity in pursuing goals and navigating society. The fifth component involves life stage outcomes or coping products, which include unfavorable or beneficial consequences associated with health, mental wellbeing, and academic achievement.

Empirical applications of PVEST demonstrate its utility for understanding Black youth development in various contexts. Velez and Spencer (2018) demonstrated the utility of PVEST as a framework for adolescent identity formation amidst converging ecological systems of inequality. Hope and Spencer (2017) explored civic engagement as an adaptive coping response to inequality conditions, examining how civic engagement serves as a proactive and reactive coping method to counteract the vulnerability and stress of systematic racial injustice. Cooper J. N. et al. (2020) and Cooper S. M. et al. (2020) applied PVEST to understand how Black fathers prepare sons to navigate racialized contexts through teaching adaptive coping skills. This study integrates intersectionality and PVEST to examine the contributions of Black male coaches to promoting positive youth development. Black male coaches occupy a unique intersectional position that allows them to serve as what we conceptualize as intersectional cultural brokers, which are individuals who leverage their own navigation of multiple marginalized identities to help youth develop adaptive coping strategies. Intersectional cultural brokering builds from previous scholarship (Nastasi et al., 2000; Lo, 2010) to conceptualize cultural brokers as those who understand their unique identities in relation to power and oppression to help advocate for the communities of their student-athletes. In this conceptualization, Black male coaches go beyond bridging the gap between their student-athletes and school community to provide system-level advocacy.

## Methods

### Research design and positionality

This study employed a qualitative interview-based approach using semi-structured interviews to explore the experiences of Black male coaches. Semi-structured interviews allow for in-depth exploration of participants’ perspectives while maintaining flexibility to pursue emerging themes and follow participants’ narratives (Braun and Clarke, 2021). This approach is particularly suited to examining how individuals understand and articulate their experiences within specific social contexts. We adopted a critical interpretive stance that acknowledges how social structures, power relations, and cultural contexts shape individual experiences and narratives, an approach aligned with both intersectionality theory and PVEST’s emphasis on person-context interactions. Our epistemological positioning is interpretive-constructivism, recognizing that knowledge is co-constructed between researchers and participants within specific sociocultural contexts (Tamminen and Dunn, 2024).

Reflexivity and transparency about the researcher’s positionality are essential in qualitative research, particularly when studying communities with which researchers share identities (Braun and Clarke, 2021). The lead author is an African American male doctoral student with experience playing competitive sports at youth and collegiate levels. His research interests are rooted in the desire to promote equitable life opportunities for the health and wellbeing of Black people across all communities. The second author is a self-described Black cisgender heterosexual male who played sports throughout his youth and is an Assistant Professor of School Psychology. The third supporting author self-describes as a Black male from the southern region of the United States for whom sports were both an outlet and an avenue to better education. All authors collaborated as a team, engaging in regular discussion to pool together collective culturally relevant knowledge that guided this study.

### Participants

This study was approved by the Institutional Review Board at the University of Kentucky. Participants were five African American males residing in different parts of the United States. Participant ages ranged from 25 to over 50 years old, with four participants between 25 and 35 years and one participant over 50 years of age. Each participant self-identified as a sports coach currently serving or having recent experience within the last 2 years serving as a baseball, basketball, soccer, or football coach at levels ranging from assistant coach to head coach. At the time of the study, one participant had obtained a Ph.D., and four coaches had a college degree or some level of college credit. Coaches worked with youth across age ranges from elementary through high school and served in various geographic contexts, including urban, suburban, and rural areas across the Northern, Southeastern, and Mid-South United States. Coaching experience ranged from 2 to over 25 years. Three participants were teacher-coaches employed by school districts, and two coaches were employed in community-based programs. All participants indicated they viewed their coaching role as extending beyond athletic instruction to include mentorship, life skills development, and support for players’ overall wellbeing. To ensure confidentiality, each participant was assigned a pseudonym, which is used in place of their name throughout this manuscript (see Table 1).

**Table 1:** Participant characteristics.

Name	Age	Sports coach	Geographical area
Terrance Mann	50+	Youth baseball at all age levels	Urban, Northern USA
Herman Boone	25–35	High school football	Urban and Rural, Southeastern USA
Mark Cooper	25–35	Girls HS and boys MS basketball	Urban, Northeastern USA
Ken Carter	25–35	Boys basketball at all levels	Rural and Suburban, Mid-south USA
James Ellis	25–35	MS wrestling, football, track	Urban, Southeastern USA

Pseudo-names were used for participants because no identifying information was collected. These are names of fictional Black male coaches from tv shows, books, and films.

### Sampling procedures

We employed purposive sampling with snowball recruitment methods appropriate for qualitative research (Braun and Clarke, 2021). Inclusion criteria specified that participants must self-identify as Black or African American male, currently serve or have served within the past 2 years as a sports coach, work primarily with Black youth or have significant experience coaching Black youth, and be willing to participate in a recorded interview. We did not exclude coaches based on specific sports, age groups served, or geographic location, recognizing that diversity across these dimensions would enrich understanding of varied coaching contexts.

Initial recruitment occurred through direct outreach via email and social media to Black male coaches with publicly available contact information. The senior author sent recruitment fliers containing study information, a Qualtrics link with key study details matching consent document information, and screening questions. Screening questions verified that coaches met the inclusion criteria by asking whether they were Black male coaches and whether they worked with Black youth. Coaches also indicated scheduling availability. After completing the screening survey, eligible participants received confirmation emails with informed consent documents and Zoom links for their scheduled interviews.

Additionally, we employed snowball sampling by asking initial participants to refer other Black male coaches who might meet study criteria. This approach proved valuable for accessing coaches in community-based settings who might not have public contact information. Snowball sampling is particularly appropriate when studying communities where insider networks and trusted referrals facilitate recruitment (Tamminen and Dunn, 2024). Our final sample of five participants aligns with recommendations for qualitative research prioritizing depth over breadth (Braun and Clarke, 2021). We assessed data saturation by monitoring whether new themes emerged during analysis and whether existing themes were sufficiently developed. After coding the fourth interview, the research team noted that no substantially new themes were emerging, and existing themes were well-developed with rich, varied examples. The fifth interview confirmed saturation, as it reinforced existing themes without introducing new conceptual categories.

### Data collection and analysis

Semi-structured interviews were conducted remotely via Zoom to accommodate participants located across different U.S. regions. Interviews lasted approximately 60 min, with a range of 45 to 75 min. All interviews were conducted by the first and third authors, who had received training in qualitative interviewing techniques. Having two interviewers present served multiple purposes as one researcher led questioning while the other took observational notes and asked follow-up questions. The interview protocol addressed four key domains: roles as coaches, resilience and coping, awareness and identification of mental health issues, supports, and barriers.

We employed thematic analysis following the codebook approach outlined by Braun and Clarke (2021). Thematic analysis is a flexible method for identifying, analyzing, and reporting patterns within data. The codebook approach involves developing a structured coding framework that can be systematically applied while remaining open to emergent codes. This hybrid deductive-inductive approach allowed us to analyze data through our theoretical frameworks while remaining open to unexpected findings. Analysis proceeded through six phases: familiarization, initial coding, theme development, reviewing and refining themes, defining and naming themes, and producing the report. Credibility was enhanced through researcher triangulation, attention to participant language, thick description, and maintaining a detailed audit trail. A second interviewer was present for real-time data collection, and a third researcher reviewed video recordings to verify question neutrality across interviews.

## Results

Through thematic analysis, we identified five major themes that illuminate how Black male coaches promote positive youth development and support youth mental health. These themes include Community Pillars, No Limits to Coaching, Relationship Fundamentals, Societal Privilege and Power Dynamics, and Struggles. These themes are interconnected and mutually reinforcing, revealing the complexity of Black male coaches’ work and their multifaceted contributions to youth and communities.

### Community Pillars

The theme of Community Pillar describes how coaches interviewed for this study are valuable assets within the Black community and leverage their position and resources to give back to the community and advocate for community needs. When asked about their current role or purpose for coaching, most participants responded that it was about giving back. Mark Cooper explained that “you’re giving back to your community, so [you] feel like [you] doing a special thing” when answering his question on what his role and purpose are as a coach. Coaching was a way for these individuals to provide the youth they work with the support they had when they were younger through positive mentoring relationships, like they had when growing up. There were a variety of different ways coaches gave back to their community, from advocating for needs to resolving racial tension to empowering children to do small acts of kindness.

When discussing how he has used his social capital as a coach to help with a community conflict, Terrance Mann provided an example from when players at his school were called racial slurs by a team they played. He described how “there was a racial incident at a soccer game and the school was keeping it quiet and really on the hush because they were playing a predominantly white school out in a rural part.” He noted that the coach would “say a little bit, but he was a status quo kind of dude. So the kids mentioned it to the parents [and the parents] were upset and they told us, you know.” This represents that parents looked to this coach to solve this issue, as he recalled “they went up there the principal ignored them”, however Terrance was able to converse with the principal and solve the conflict.

Aside from resolving racial tension within their community, all coaches in this study had some type of service program that they and their children participated in throughout the year. Ken Carter reported that “they don’t know anything about community service so when we go out and rake leaves, they like oh we about to go make money, I said no this is for free we’re doing it for elderly people, people who can’t rake their leaves, so we go out and we do it for free”. These programs teach players how they can give back through small tasks. He later reported that the children felt “so gratif[ied] like afterward when it’ll be a parent or whoever we raked the leaves for, they would be like man I’m so thankful that you guys came nobody would come, and I could see how much [the kids] got from that”. Additionally, coaches hosted community events where they would post flyers and contact parents, and meet at gyms and community events for pick-up games, athletic development, and community gathering. Coaches in this study used facilities at their school to host year-round workouts and community gatherings. These activities position coaches as community organizers, creating spaces for connection and collective support.

### (No) Limits to Coaching

Participants were asked specifically about their roles and responsibilities as coaches, whether they felt there were limits to what they could do, and how well they felt they could help students and players. While responses varied based on specific situations and contexts, most coaches in our study reported that there were no limits to how supportive they could be to players and their players’ families. This theme is opened with a lengthy yet critical quote from Ken Carter that best exemplifies how sports and coaching serve as avenues to understanding life.

Ken Carter explained that “[I have] a kid now whose repeated behavior is to dribble the ball into the corner and pick it up. In basketball you do not go to the corner and pick the ball up because a trap is coming. People get these signs in life that kind of point them in certain directions and a lot of people turn a blind eye to them and do not pay them any attention, but they are signs in life.” Ken Carter reflects on conversations with players where they process these signals together which sparks realizations, saying “I probably shouldn’t hang with this person or if I hang with this person it’s probably gonna draw this kind of crowd, I probably shouldn’t talk to this person because they are toxic, I probably shouldn’t skip school because I don’t know what may happen. So people get those signs in life like in basketball if you go to the corner and do not see any of your teammates there you are probably in a bad spot, so you might want to get out of there or get rid of the ball.” Carter discusses “we like to dribble through traps and I always talk about how we gamble so much, but we don’t get paid, so in life, you know you have these these moments where you taking risks and this goes back to the consequences in decisions, so if you if you try to split a defense with the ball or in practice you try to do something extra that’s not necessary. You may or may not make it through, that’s a life gamble that you’re taking…So what I always tell them is, man, everybody’s not fortunate enough to get a second chance. Some people can do the same stuff, you can take the same drug, I can overdose and you could not, you can drink the same amount of drinks, I could get alcohol poisoning.”

Ken Carter said that he often communicates how situations in sports replicate aspects of encounters youth may have in life. Black youth, especially those who have been disenfranchised, have to face the difficulties of learning how to navigate dealing with life’s hardships at an early age. These coaches take the opportunity to teach the children about life stressors to help them better prepare for future games and life encounters that will test their physical and mental strength, as well as seize the opportunity to peer into the personal lives of the kids with whom they work. Mark Cooper uses an exercise called “time and score” to help players learn how well they can work with others during stressful situations. He explained that “they have to make it down the court to score, and it took 25 seconds, and they work on how players handle this pressure. They have tried to pump a little crowd noise for YouTube over the speakers to see what their resilience is like, what their attitude is, and how they talk to their teammates during this critical situation.” He noted that “they want to always be thinking critically and highly and always thinking next play because that is what it is in life, and when something does not go your way you move on to the next play, the next thing, the next day.”

These examples illustrate coaches using sport as what we term developmental laboratories, which are intentionally structured environments where youth can practice life skills in relatively safe contexts before encountering higher-stakes situations. Beyond abstract analogies, coaches engaged in more direct interventions. Nearly all participants mentioned they have year-round programs in which they teach children topics related to dress attire, etiquette, dealing with law enforcement, and eating healthy. The coaches themselves emphasized that they take the opportunity of their limitless role to notice when their players show signs of sadness or depression and try to remedy the difficult times their players are dealing with by being someone they can relate to and talk to about what is going on in their lives, whether at home, school, or in the community.

James Ellis described how he is present to support players’ needs: “[I want] to help them deal with whatever is going on, and… that is why it leads to high percentages and suicide amongst young Black men especially in certain age ranges. [I want] to help break that stereotype showing that it is okay to let it out and if you need to talk about something or seek help with something, that is okay… [asking] how players feel as a way of showing that as a Black man [I] can care about another Black man and his mental health and emotional health as well.” Significantly, all coaches in this study were aware of the growing suicide rate among Black youth and brought it up before questions related to mental health were asked. Regardless of whether they felt prepared to have these conversations, the relationships they have with their players facilitate dialogue on various topics related to challenges that go beyond sports.

The limitless role that these coaches described is evidenced by testimony from Mark Cooper about a previous coach he worked with, noting that “[I] had a coach at another school basically adopt a kid who was in a bad situation and that kid is basically like their son now.” Richardson’s (2012) conceptualization of coaches as social fathers is fully embodied here, with coaches also serving as support for parents by mediating conversations and problems between children and parents. Herman Boone mentioned that coaches are sometimes the very first contact for parents when something is not working out with their children. It was common for these coaches to share how they have seen coaches act as great contributors to struggling kids and their families by paying light bills, driving kids back and forth to practice, or even taking on the caregiver role.

Mark Cooper noted limitations he faces as a Black man coaching women, stating that “you know I’m coaching women, so I feel like there’s a limit, with a man coaching women but guys coaching boys, I feel like there is no limit” Mark Cooper was the only participant in this study who primarily coached women. Herman Boone mentioned another source of limitations related to volunteer coaches versus teacher-coaches, noting that a coach who volunteers has no limitation as far as how to go against the rules or regulations of being a teacher or professional. Both examples showed that demographic characteristics like gender and professional role play critical roles in how one navigates relationships with children to coach them.

### Relationship Fundamentals

Relationship Fundamentals are the building blocks of relationships between coaches and players. These building blocks are valuable qualities of any relationship, and coaches in this study model and foster development of traits like authenticity, accountability, dedication, self-reflection, trust, and communication in a manner that warrants reciprocity from their players. As mentioned by James Ellis, “these kids can tell when you are not authentic and it is the pathway to building relationships.” James Ellis coaches mostly middle school sports, and this age group needs authentic exposure to positive representations of Black men who are authentic to themselves to aid with their identity development.

Trust is also a building block for positive relationships with youth. Ken Carter provided an example of how critical trust is to meet the needs of his players: “like no everything is good’,[I’m] like, ‘you kidding?’, but then I see that the trust hasn’t been built with that person yet so even if it’s [a mental health] professional, even if it’s a preacher or if it’s a businessman, the trust is not built. The kid is not going to reach out and say hey I’m dealing with this and I’m dealing with that, there has to be trust, [and] some kind of foundation built there so someone may say ‘hey coach I feel more comfortable with you than I feel [talking] to my mom.’”

Ken Carter and James Ellis provide insight into the importance of these qualities, suggesting that other professionals and caregivers may be able to use Relationship Fundamentals to make themselves available, comfortable, and accessible to students. The trust established can also help players feel comfortable with vulnerability with their coach. Ken Carter illustrates that trust, vulnerability, and accountability are rehearsed through sports. Carter states “The errors players make, they kind of know why, so what that creates is accountability, and when those errors happen in a game players say my bad coach, so the same thing happens in life.” The relationship through sports helps players model what it is like to quickly reflect on their actions, take ownership of mistakes, communicate with someone they trust, and move forward.

Lastly, coaches in this study all mentioned meeting children where they are when it comes to communication and talking to them. James Ellis, Ken Carter, Herman Boone, and Mark Cooper all mentioned strategies for talking to youth daily during various routines that are optimal for dialogue with particular players. Daily routine communication occurred as wellness check-ins during meals, after playing games, or in Terrance Mann’s case while in the dugout. Not only is frequency important but also communication style. As Herman Boone stated, “some kids you might not have to talk to as aggressive because they are already more straight line or guidance is needed in other ways, while some kids are quote-unquote knuckleheads and that is the only thing that they kind of respond to.” These coaches take time to learn from their players and understand when and how to engage with each one for optimal communication.

### Societal Privilege and Power Dynamics

During discussions of life stressors that uniquely affect the youth they work with, coaches’ responses indicated they are all aware of societal issues that create power dynamics Black youth must navigate safely. These included aspects such as racism, gentrification, police, exploitation, and being affected by violence within society. It was important to all participants that they share stories to acknowledge these issues and give time for children to process with them. Related to this theme was the overwhelming lack of awareness of societal exploitation of Black youth and lack of importance placed on that awareness.

Terrance Mann shared how he has seen this happen to many kids, but he specifically knew of a kid who was a good athlete but got into some trouble during the transition from middle school to high school. He recounts that “they got to high school and the coach used the brother’s body up but was not really trying to help him get through school, so he kind of fell by the wayside and nobody was really checking for him like that, and he did not fulfill the potential that he really had.” Herman Boone added that the community plays a role in this exploitation as well. He asks “how many times have you seen that guy as soon as he is done entertaining people on Friday night and he is really out there struggling in the real world, where is all that same support he was getting? the same way people were willing to help or be there during game time, somebody in the community could have opened up or gave opportunities to help change his life.” Later in the interview, Herman Boone emphasized that coaches have to challenge the community to support players not just during their playing days but later on to keep that same support when they graduate.

One mistake can be costly for some children. As Terrance Mann discussed in his comments on the (de)valuing of Black boys, he stated that “so many people throw these Black boys away. Once a mistake is made and kids are no longer useful for their athletic production, they get thrown away with nobody coming to check on them, uplift them, teach them how to navigate life and make better decisions, preventing them from reaching their full potential.” This is why Terrance Mann, as well as all coaches in this study, emphasized placing great importance on building sustainable relationships beyond sports and using sports to open up opportunities for positive life outcomes while they are playing. While coaches acknowledge that players may feel privileged while playing, they recognize youth can concurrently be exploited and after they finish playing, be discarded.

### Struggles

The last common theme concerned struggles and barriers for these Black coaches to work as effectively as possible with their players, families, and community. All participants noted the evolution of technology for communication and social media as issues that hindered their ability to support children. Herman Boone spoke about “social media being a distraction, “with instant gratification, people make a documentary just to say they are transferring from one school to another, like c’mon man you’re losing the essence there.” Social media is another aspect that coaches now invest in to support youth concerning issues of players pursuing viral attention and monitoring peer relationships. Ken Carter finds this distracting during games, noting that “some players don’t even want to contest with a shot because they do not want the kid to do the three finger gesture to the head after making the shot.”

Coaches shared their experience with family systems as participants demonstrated ability to mediate issues between children and parents but also support when children’s needs are not fully understood by parents. Ken Carter recalled experience navigating a child crisis with a family:

“[I] always ha[ve] a group chat with the kids and one night the group chat was going and they were having a really rough night and one kid was talking about killing himself and talking really weird. A couple parents were saying they have a one year old and can they please tone it down a bit, and [I] said… now is not the time to worry about sleep and if it is a problem to mute the phone because they have a kid talking about possibly harming himself and they really want to address that. So I got in touch with the kid’s people and they said that was just one of his attention seeking methods and a lot of times they ignore that”, but Ken Carter noted “that is one of those things you can never ignore and you have to take the seriousness of someone’s threat.”

Not only did coaches face challenges from parents who may not accept mental health as a priority for their child, but some sports programs are run by administrators and head coaches who are barriers for participants to promote positive mental health and provide spaces where children can be vulnerable about their challenges. Mark Cooper stated, “[I] feel[s] like the barrier sometimes is that people don’t have that support and coaches do not have support from administration. AD’s and other coaches on staff can be barriers, and if the head coach doesn’t want you to build a relationship or if the AD says they are not doing this with the players, if you do not have that support to do things outside of sports you are really stuck in quicksand and cannot really build.”

Herman Boone also commented on the idea of a program’s priority being sports only, noting that “I’ve been at some sports programs where ADs and head coaches don’t allow space to build relationships outside of sports. They say ‘aye this is my show, keep it what it is, and keep it going… [you] wanna say how you feel you better go talk to somebody else, don’t come talk to me about it…we’re really just coaching players’ and you can’t really do just coach players you know you.” Coaching a player provides a critical opportunity for these individuals to build enduring trusting relationships with students that can help them be resilient as they develop through adolescence into adulthood. These threats to their ability to build relationships ultimately prevent providing youth with outlets to express themselves and communicate their needs to adults who are responsible for their development and growth.

## Discussion

To date, the literature on the role of Black male coaches has not been fully explored. This study is foundational to understanding the roles Black male coaches play in the positive development of Black youth by providing analysis of interviews with five Black male coaches serving youth across different regions, sports, and contexts in the United States. This study was conducted to address gaps in the literature since Richardson’s (2012) pioneering study and to bridge gaps by applying theoretical frameworks that center the intersectional experiences of Black male coaches and their assets to youth development using intersectionality and PVEST frameworks.

Our findings that Black male coaches serve as community advocates, conflict resolvers, and organizers extend Richardson’s (2012) conceptualization of coaches as social capital. While Richardson documented coaches’ roles as social fathers and community resources, our findings provide additional specificity about mechanisms through which coaches leverage their positioning. Coaches in our study advocated for players experiencing racism, organized community service activities that modeled civic engagement, and created year-round programming extending far beyond athletics. These practices align with Thomas’s (2022) documentation of Black male teacher-coaches’ civic-oriented thought and practices. Thomas (2022) found that Black male teacher-coaches view their work as inherently connected to community uplift and social justice, using their positions to address inequities and empower youth. Our findings suggest this orientation extends to community-based coaches as well, not just those employed by schools.

This has important implications for understanding Black male coaches as what we term intersectional cultural brokers, which are individuals positioned at intersections of multiple communities including sports, education, and the Black community who leverage their positioning to advocate, connect, and mobilize resources for youth. From a PVEST perspective, coaches’ community advocacy work operates at the contextual level, attempting to reduce risk contributors including racism and resource scarcity and create more supportive developmental ecologies. By addressing incidents of racism directly, organizing community support systems, and modeling civic engagement, coaches work to transform the very contexts in which youth development occurs.

The theme No Limits to Coaching resonates with research on effective positive youth development programs emphasizing explicit life skills instruction and intentional transfer activities (Holt et al., 2017). Coaches in our study used sport as what we term as developmental laboratories, which are structured environments for practicing skills applicable to life contexts. This practice-based approach aligns with research finding that life skill development is most effective when it is intentional rather than assumed to occur automatically through sport participation (Hart, 2020; Williams, 2023). Our findings provide examples of how Black male coaches specifically employ these strategies, including using practice scenarios to build stress management skills and conducting year-round programming on topics like interacting with law enforcement, which are issues particularly salient for Black youth navigating racialized systems.

Significantly, coaches’ awareness of and willingness to address mental health concerns, particularly suicide risk, represents a critical contribution given the Black youth mental health crisis (Bridge et al., 2018; Sheftall et al., 2022). All coaches in our study mentioned suicide risk unprompted, demonstrating awareness of this issue. Their openness to having mental health conversations positions them as potential partners in suicide prevention efforts, aligning with Congressional Black Caucus Emergency Task Force on Black Youth Suicide and Mental Health (2019) recommendations for community-based approaches. The concept of coaches as social fathers, exemplified by coaches paying bills, transporting youth, and even adopting players, powerfully illustrates the depth of commitment these men bring to their work. This goes beyond traditional mentoring to what might be conceptualized as fictive kinship, which refers to family-like bonds formed outside biological relationships that are particularly important in Black communities (Richardson, 2012).

Our findings on Relationship Fundamentals align with coach-athlete relationship research emphasizing trust, communication, and authenticity as foundational to quality relationships (Jowett, 2017). The practices coaches described including individualized communication, modeling vulnerability, and meeting youth where they are reflect core components of Jowett’s 3 + 1Cs model. Importantly, our findings extend this literature by illuminating how Black male coaches specifically build trust with Black youth who may have experienced community, institutional betrayal, and systemic harm. Coaches emphasized that professional credentials matter less than authentic connection and cultural understanding for establishing trust. Black male coaches’ insider status, sharing racial and often gender, class, and community identities with youth, may facilitate trust-building in ways that professionals without these shared identities cannot replicate.

The theme Societal Privilege and Power Dynamics demonstrates coaches’ critical awareness of how Black youth are simultaneously valued for athletic production yet devalued as human beings deserving holistic support. This finding aligns with scholarship on structural racism in education and athletics documenting how institutions extract labor and performance from Black bodies while denying educational opportunities and resources (Alvarez et al., 2022). Coaches’ recognition of these dynamics positions them to help youth develop critical consciousness about oppressive systems while building skills to navigate them, consistent with PVEST’s emphasis on adaptive coping within contexts of systemic risk. The Struggles theme illuminates significant barriers limiting coaches’ effectiveness. The barriers coaches identified reflect what Bristol (2020) conceptualizes as boundary-heightening experiences, which are organizational constraints that limit Black male educators’ abilities to enact their visions for supporting youth.

## Theoretical contributions

This study extends application of PVEST (Spencer et al., 1997) to understanding Black male coaches’ work. Coaches in our study addressed all five PVEST components in their practice. Regarding risk contributors, coaches demonstrated awareness of systemic inequity, poverty, violence, and exploitation that Black youth face. They directly addressed these risks through advocacy by confronting racialized incidents, resource provision through community service and programming, and critical consciousness development. Through stress engagement, coaches recognized signs of stress in youth including behavioral changes and emotional struggles and created spaces for youth to process stress. Their awareness of suicide risk and willingness to address mental health represents direct engagement with this component.

Regarding reactive coping methods, in developmental laboratories, coaches helped youth practice coping strategies including stress management during timed exercises, accepting accountability after mistakes, and engaging in communication under pressure. They modeled both effective and ineffective coping while explicitly teaching the difference. Culminating in stable coping responses and emergent identities, coaches supported racial identity development by modeling positive Black masculinity, teaching about racism, and affirming cultural pride. Community service and civic engagement activities promoted identities as community members with agency to create change. Coaches’ holistic focus on academics, life skills, mental health, and character alongside athletics aimed to promote positive life stage outcomes. Importantly, our findings suggest that effective Black male coaches do not merely support youth navigating PVEST components but actively intervene at ecological levels to reduce risk contributors and strengthen protective environments.

This study demonstrates intersectionality’s (Crenshaw, 1998) value for understanding Black male coaches’ experiences and practices. Coaches’ intersectional positioning as Black men in predominantly White sports institutions created both challenges including navigating racism and proving competence and opportunities including cultural connection with Black youth and insider knowledge of community needs. Their intersectional identities shaped what we termed intersectional cultural brokering, which refers to leveraging multiple community connections to advocate for youth, connect them to resources, and help them navigate complex social terrain. Coaches’ awareness of how intersecting systems, including racism, classism, and athletic exploitation, shape youth experiences reflects sophisticated intersectional analysis. We introduced concepts of developmental laboratories, counter-exploitative coaching, and fictive kinship to guide future research and theory development.

## Implications for Black psychology

This research makes several contributions to Black psychology, a field dedicated to understanding the psychological functioning of African Americans and promoting liberation, wellbeing, and empowerment (Burkett, 2017). In centering community strengths, rather than deficit-focused approaches emphasizing what Black communities lack, this research centers assets Black communities possess, specifically Black male coaches as cultural resources who promote development, support mental health, and strengthen communities. This strengths-based framing aligns with African-centered psychology’s emphasis on resilience, cultural assets, and collective empowerment. Nobles (2015) articulated the importance of moving from Black psychology to Sakhu Djaer, emphasizing the ongoing development of a Pan-African Black psychology that centers African philosophical traditions and community strengths.

This study’s findings illuminate specific protective factors within Black communities that buffer youth from risk and promote positive development. These include access to Black male mentors and role models, culturally grounded relationships emphasizing authenticity and communalism, critical consciousness about systemic oppression combined with skill-building for navigation, and community-based support networks extending beyond formal services. Understanding these protective factors can inform prevention and intervention development. Yosso’s (2005) community cultural wealth framework provides additional theoretical grounding for understanding the multiple forms of capital that Black male coaches embody and transmit to youth.

This research contributes to understanding community-based approaches to the Black youth mental health crisis. Coaches’ awareness of suicide risk, willingness to address mental health, and trusted relationships with youth position them as potential partners in suicide prevention efforts. Their community embeddedness may allow access to youth who will not engage with formal services due to stigma or mistrust. Coaches’ practices reflect culturally grounded approaches, including communal orientations, extended family structures through fictive kinship, collective uplift through community service, and recognition of systemic barriers alongside individual agency. These practices align with Afrocentric values and may be more culturally congruent for Black youth than Eurocentric intervention approaches. Utsey et al. (2001) articulated a psychology of liberation for counseling African Americans confronting societal racism and oppression, providing additional theoretical grounding for understanding coaches’ liberatory practices.

## Limitations and future directions

Limitations should be considered when interpreting findings. Our sample of five Black male coaches, while appropriate for qualitative research prioritizing depth, limits the breadth of experiences captured. All participants were cisgender heterosexual Black men, and future research should include coaches with diverse intersectional identities. Only one coach in our sample primarily coached girls and young women, limiting understanding of gender dynamics in coach-athlete relationships.

Qualitative research using semi-structured interviews prioritizes understanding participants’ experiences and perspectives in depth rather than measuring outcomes or establishing causality.

While we documented coaches’ practices and perspectives, we did not collect data from youth, families, or communities about coaches’ impacts. Future research should include multi-perspective designs examining youth outcomes associated with different coaching approaches. Building on study limitations and findings, we identify several priority directions for future research, including intersectional analyses examining how multiple identities shape coaching, youth perspectives on Black male coaches’ contributions, organizational factors supporting or constraining holistic approaches, coach training development, and community-based participatory research engaging coaches, youth, families, and communities as co-researchers.

## Conclusion

This qualitative study employing semi-structured interviews explored how Black male coaches promote positive youth development and support mental health among Black youth. Through semi-structured interviews with five Black male coaches, we identified five interconnected themes including Community Pillars describing coaches as advocates, organizers, and cultural assets, No Limits to Coaching describing holistic roles extending far beyond athletics, Relationship Fundamentals describing trust, authenticity, and culturally grounded connections, Societal Privilege and Power Dynamics describing critical awareness of systemic exploitation combined with counter-exploitative practices, and Struggles describing barriers including technology challenges, family dynamics, and organizational constraints.

Our findings demonstrate that Black male coaches possess significant social capital within Black communities, functioning as social fathers, mental health supporters, community advocates, and cultural brokers. They leverage their intersectional positioning, sharing racial and often gender and class identities with youth while navigating predominantly White sports institutions, to build trusting relationships and promote holistic development. Through intentional pedagogical practices, they use sports as developmental laboratories where youth practice life skills, develop adaptive coping strategies, build racial identity, and access support networks. Importantly, coaches demonstrated awareness of the Black youth mental health crisis, particularly rising suicide rates, and willingness to address mental health concerns despite limited formal training. Their community embeddedness and trusted relationships position them as potential partners in community-based suicide prevention efforts.

Schools and athletic departments should recognize and support coaches’ multifaceted roles beyond winning games, providing time and resources for relationship-building activities, professional development in trauma-informed practice in mental health support, and evaluation criteria valuing holistic youth development. Mental health professionals working with Black youth should recognize and partner with Black male coaches as trusted community resources. Community organizations should invest in recruiting, supporting, and retaining Black male coaches. Black male coaches represent what we term as intersectional cultural brokers, individuals positioned at intersections of multiple communities who leverage their lived experiences navigating marginalized identities to support youth facing similar challenges. This study further illustrates the need for more conceptual models of childhood resilience to include Black male coaches alongside parents, teachers, and other stakeholders. As we address the alarming crisis of Black youth suicide and mental health disparities, Black male coaches should be recognized and supported as vital partners in community-based prevention and intervention efforts.

## Data Availability

The original contributions presented in the study are included in the article/supplementary material, further inquiries can be directed to the corresponding author.
